# Variations in bacterial and archaeal communities along depth profiles of Alaskan soil cores

**DOI:** 10.1038/s41598-017-18777-x

**Published:** 2018-01-11

**Authors:** Binu Mani Tripathi, Mincheol Kim, Yongwon Kim, Eunji Byun, Ji-Woong Yang, Jinho Ahn, Yoo Kyung Lee

**Affiliations:** 1 Korea Polar Research Institute, Incheon, 21990 Republic of Korea; 2 International Arctic Research Center, University of Alaska, Fairbanks, Alaska USA; 3 Present Address: School of Earth and Environmental Sciences, Seoul National University, Seoul, Republic of Korea; 4 Department of Earth Sciences,, University of Toronto, ON, Canada

## Abstract

Understating the microbial communities and ecological processes that influence their structure in permafrost soils is crucial for predicting the consequences of climate change. In this study we investigated the bacterial and archaeal communities along depth profiles of four soil cores collected across Alaska. The bacterial and archaeal diversity (amplicon sequencing) overall decreased along the soil depth but the depth-wise pattern of their abundances (qPCR) varied by sites. The community structure of bacteria and archaea displayed site-specific pattern, with a greater role of soil geochemical characteristics rather than soil depth. In particular, we found significant positive correlations between methane trapped in cores and relative abundance of methanogenic archaeal genera, indicating a strong association between microbial activity and methane production in subsurface soils. We observed that bacterial phylogenetic community assembly tended to be more clustered in surface soils than in deeper soils. Analyses of phylogenetic community turnover among depth profiles across cores indicated that the relative influence of deterministic and stochastic processes was mainly determined by soil properties rather than depth. Overall, our findings emphasize that the vertical distributions of bacterial and archaeal communities in permafrost soils are to a large extent determined by the variation in site-specific soil properties.

## Introduction

Approximately 25% of Earth’s terrestrial surface is underlain by permafrost^1^, which encompasses ∼50% of the total global soil carbon (~1672 Pg;^2,3^). However, permafrost soils are warming rapidly due to global climate change^4^, potentially resulting in extensive thaw and increased thickness of the seasonally thawed soil active layer. The thawing of the permafrost soils will expose frozen carbon pool to microbial decomposition^5,6^, which could further enhance the emission of carbon dioxide (CO_2_) and methane (CH_4_) from the soil, and potentially generate a positive feedback to climate warming^7^.

Despite prolonged subzero temperature and low water availability, a diverse group of microorganisms inhabit permafrost soils^8–11^. In a broader biogeography study, the Arctic tundra surface soils showed similar levels of bacterial diversity with soils of other biomes^12^. Microbial communities were also compared between active and permafrost soil layers and both layers displayed highly similar phylogenetic and functional community composition^13^. The majority of the microorganisms present in permafrost soils are cold-adapted heterotrophs belonging to the bacterial phyla such as *Proteobacteria*, *Actinobacteria*, *Acidobacteria*, *Firmicutes*, *Bacteroidetes*, and *Chloroflexi*, while others belong to archaeal phyla *Euryarchaeota*, *Crenarchaeota*, and *Thaumarchaeota* ^14^. However, it is still not well understood how this complex active and permafrost soil layer microbial community is influenced by various environmental and ecological factors.

Understanding the factors influencing the assembly of soil microorganisms along the permafrost thaw gradient is critical to predict the potential consequences of climate change^1^. In recent years, several studies have reported the depth specific profile of microbial communities along permafrost thaw gradient and revealed that microbial community compositions are influenced by a number of abiotic factors such as soil pH^15^, moisture^16^, conductivity^17^, soil organic matter quality^18^, and nutrient availability^19–21^. However, it is still not clear how the microbial community composition and diversity would vary along the permafrost thaw gradient sampled across different ecosystems.

Deterministic and stochastic processes are known to influence the assembly of microbial species in a local community^22–24^. Recent studies have suggested that rather than acting independently, both deterministic and stochastic processes interact to shape microbial community assembly^25–27^. The relative influence of deterministic and stochastic processes in shaping microbial community assembly is examined in various environments^25,26,28–30^, except in terrestrial cryoenvironments, which have been relatively poorly studied^31^. Thus, there is need to study the microbial community assembly and underlying ecological processes along the permafrost thaw gradient from different ecosystems.

We sampled four soil cores with different vegetation covers in Alaska to examine bacterial and archaeal communities along active layer and permafrost thaw gradient. This study was set out to answer the following questions:

1. How do the abundance, composition and diversity of prokaryotic community vary along the depth in permafrost soils?
2. Are there any bacterial or archaeal taxa specific to sampled sites or soil layers of the cores? Are those taxa are associated with unique geochemistry or trapped gases of the cores?
3. What are the major environmental factors that influence the assembly of prokaryotic community along the depth?
4. How does the relative influence of deterministic and stochastic processes vary along the depth to influence the assembly of prokaryotic community?

## Materials and Methods

### Site description and soil core sampling

**Figure 1:**
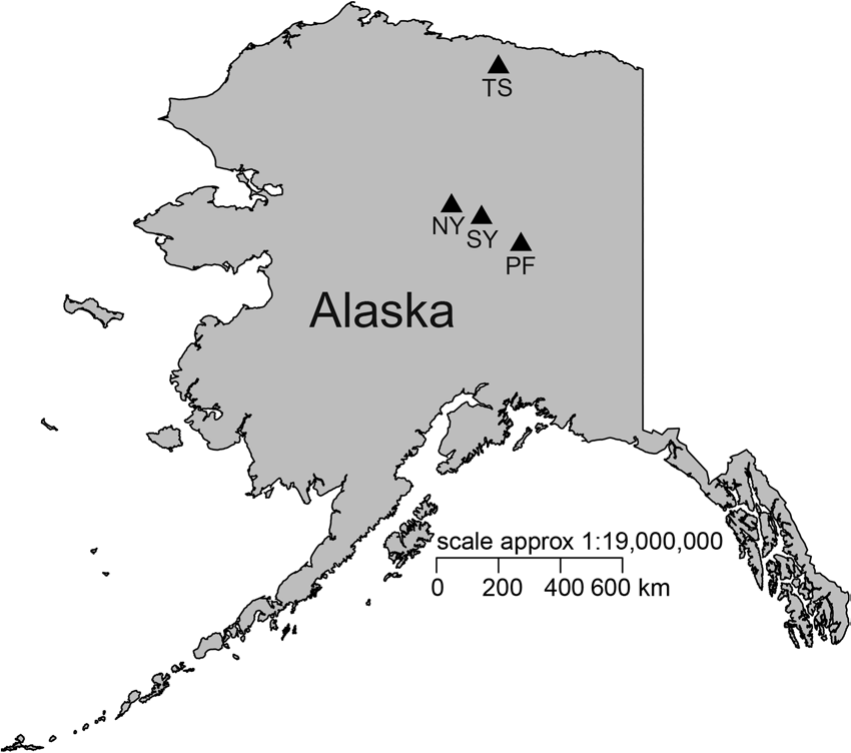
Soil core sampling site locations along the haul road of Alaska. The map was generated in R software version 3.2.3^51^ using package ‘mapdata’^78^ (https://cran.rproject.org/web/packages/mapdata/index.html). TS = tundra near Sag River; NY = north of Yukon River; SY = south of Yukon River; PF = Poker Flat Research Range.

Four different sites were selected for this study located along the trans-Alaskan pipeline (Fig. 1). These sites were one in tundra near Sag River (TS), two were in black spruce boreal forest towards north and south of Yukon River (NY and SY), and the other one was also in a black spruce forest in interior Alaska dominated by Sphagnum moss at Poker Flat Research Range (PF). Except TS which was located in continuous permafrost zone, the other three sites were located within discontinuous permafrost zone (see^32^ for detailed site descriptions).

Field sampling was carried out during the early spring in the months of April to May 2013. Soil cores were collected using a SIPRE coring auger (3-inches in diameter with PVC liners fitted in the auger). The TS, NY and SY sites were completely frozen from surface to bottom during the time of drilling, whereas the top 20–30 cm soil layer of PF site was thawed. All the four cores were sealed in PVC liners (90 cm long and 7.6 cm in diameter) and shipped to the laboratory in frozen state (−20 °C). A total of 42 samples were collected from four cores for further analysis, with 8–13 samples taken along the depth from each core.

### Soil properties and trapped greenhouse gas analysis

Soil pH and electrical conductivity (EC) were measured in a soil-water suspension (1:5 ratio, w/v) using a pH/EC benchtop meter (Orion Star A215, Thermo Scientific, Waltham, MA, USA). Gravimetric water content (GWC) was measured by weighing the soils before and after drying at 105 °C for 12 h. Total organic carbon (TOC) and total nitrogen (TN) contents were measured using an elemental analyzer (FlashEA 1112 Thermo Electron corporation, Waltham, Massachusetts, USA) at the National Instrumentation Center for Environmental Management (NICEM, Seoul, Korea).

Soil core temperature was measured from boreholes drilled into the surface at 80 to 90 cm depths since 2013 to now using custom-made temperature profilers, consisting of 12 temperature sensors (TMC20-HD, Onset Comp., USA) and three data loggers (U12-008, Onset Comp., USA). The trapped CH_4_ and CO_2_ in frozen soil samples were analyzed using a headspace gas extraction method^33^. The frozen soil samples collected along the depth of each core were taken in a 40 ml sampling vials and filled with 10 ml 6 M NaCl solution. The sampling vials were sealed and heated at 65 °C for 30 minutes to extract the headspace air. The CH_4_ and CO_2_ concentrations of headspace air were measured by injecting 1 ml gas into an Agilent Technologies 7890 A gas chromatograph with flame ionization (FID) and thermal conductivity detectors (TCD) (Agilent Tech, Santa Clara, CA, USA) at the Korea Institute of Geoscience and Mineral Resources (KIGAM, Daejeon, Korea). The methane and carbon dioxide mixing ratios were calculated from peak areas by comparing with several standard gases (10.2, 100, 1050 ppm, 1%, 10% for CH_4_, and 1%, 10% for CO_2_). Total uncertainties for the whole procedure were better than 6.9% for CH_4_ and 6.2% for CO_2_. All the measurements of soil properties and trapped greenhouse gases were performed previously by Byun *et al*.^32^.

### DNA extraction, PCR amplification and sequencing

For each sample, a single DNA extraction was performed on 0.50 g of soil using FastDNA^TM^ SPIN Kit for Soil (MP Biomedicals, Santa Ana, CA, USA) according to the manufacturer’s protocol. The primer pair Bakt_341F (5′-CCTACGGGNGGCWGCAG-3′) and Bakt_805R (5′-GACTACHVGGGTATCTAATCC-3′) was used to amplify the V3 and V4 region of bacterial 16 S rRNA gene^34^. Whereas, the V3 region of archaeal 16 S rRNA gene was amplified using the primer pair S-D-Arch-0349-a-S-17 (5′-GYGCASCAGKCGMGAAW-3′) and S-D-Arch-0519-a-A-16 (5′-TTACCGCGGCKGCTG-3′)^35^. The resulting amplicons were sequenced using paired-end (2 × 300 bp) Illumina MiSeq system (Illumina,USA) at Macrogen Incorporation (Seoul, Korea).

### Sequence Processing

The pair end sequences were trimmed based on quality scores with Sickle (https://github.com/najoshi/sickle) followed by error correction with BayesHammer^36^.

The resulting quality trimmed and error corrected paired-end sequences were assembled using PANDAseq software^37^. This approach has been shown to reduce the substitution error rates by ~93% in Illumina MiSeq sequence data^38^. Further sequence processing steps were performed on mothur pipeline^39^. The assembled sequences were aligned against a SILVA alignment (http://www.arb-silva.de/), and subsequently denoised using ‘pre.cluster’ command implemented in mothur. Chimeric sequences were removed using mothur’s command ‘chimera.uchime’ in *de novo* mode^40^. The bacterial and archaeal 16 S rRNA gene sequences were taxonomically classified against EzTaxon-extended database^41^, using the naïve Bayesian classifier implemented in mothur (at 80% bootstrap cutoff with 1000 iterations). The sequences were further clustered to operational taxonomic units (OTUs) at 97% sequence similarity level using the average neighbor algorithm implemented in mothur. The entire singleton OTUs were removed prior to statistical analysis.

Despite using Archaea-specific primer set, we detected a lot of bacterial reads in each sample, which resulted in highly variable archaeal sequence reads across samples (120–6495 reads). Therefore, we only performed community composition analyses on archaeal sequences. For bacterial sequences, we analyzed the composition, diversity and assembly processes with rarified sequence data (2,453 reads per sample). All the sequence data used in this study are deposited to the Sequence Read Archive (SRA) at NCBI under the accession number SRP124819.

### Quantitative PCR analysis

We performed quantitative PCR (qPCR) using CFX96 qPCR System (Bio-Rad, Hercules, CA) with SYBR Green as reporter dye (Bio-Rad, USA) to estimate the 16 S rRNA gene abundance of bacterial and archaea. Partial 16 S rRNA genes were amplified using lineage-specific primer pairs targeting bacteria (341 F: 5′-CCTACGGGAGGCAGCAG-3′)/797 R: 5′-GGACTACCAGGGTCTAATCCTGTT-3′^42,43^,) and archaea (Arch349F: 5′-GYGCASCAGKCGMGAAW-3′/Arch806R(5′-GGACTACVSGGGTATCTAAT-3′^35^,). For each sample, the qPCR reaction was carried out in duplicates for 40 cycles with denaturation at 94 °C for 25 s, primer annealing for bacteria at 50 °C and for archaea at 50 °C for 25 s, and extension at 72 °C, each for 25 s. A tenfold dilution series of plasmids containing the bacterial and archaeal 16 S rRNA gene from soil samples were used as the standard curves.

### Phylogenetic analysis

A maximum-likelihood tree was constructed with aligned bacterial 16 S rRNA gene sequences using FastTree program with default settings^44^. Standardized effect size measure of mean nearest taxon distance (SES.MNTD) was calculated using the null model ‘taxa.labels’ (999 randomization) in Picante R package^45^. The lower magnitude of SES.MNTD values (<0) indicate phylogenetic clustering, whereas higher magnitude of SES.MNTD values (>0) indicate phylogenetic over dispersion^46^. Turnover in phylogenetic community composition was quantified using β-mean nearest taxon distance (βMNTD) in Picante R package (‘comdistnt’ function), which is between-community analog of MNTD. Furthermore, to infer the relative influence of community assembly processes, we calculated β-nearest taxon index (βNTI) which is referred as difference between observed and mean of null distribution of βMNTD normalized by its standard deviation. The pairwise comparisons with βNTI values <−2 and >+2 indicate deterministic assembly with less than (homogenous selection) and more than (variable selection) expected phylogenetic turnover^28^, respectively.

### Statistical analysis

The correlation matrix of soil properties data was used to perform principal components analysis (PCA) in Canoco 5.0 (Biometrics, Wageningen, The Netherlands). Permutational multivariate analysis of variance (PerMANOVA, ‘adonis’ function in vegan R package^47^) was used to test the effect of sampling site on soil properties data (Euclidean distance matrix of normalized data) with 9999 random permutations.

Non-metric multi-dimensional scaling (NMDS) ordinations were used to visualize the pairwise Bray–Curtis dissimilarity matrices. We used PerMANOVA to evaluate the effect of sampling site on Bray–Curtis dissimilarity matrices (9999 random permutations). To examine the association between community structure and soil properties, the vectors of significant soil properties were fitted onto NMDS ordination space by using ‘envfit’ function of vegan R package. Redundancy analysis (RDA)-based variation partitioning analysis^35^ was performed to assess the relative influence of soil depth, sampling site, and soil chemical properties on composition of bacterial and archaeal community. We used Hellinger-transformed OTU abundance table as response variable for variation partitioning analysis.

We performed linear regression analysis to analyze the relationship between alpha-diversity (Shannon index) and depth. Furthermore, liner mixed effects model was used to investigate the effect of soil properties on Shannon diversity index using ‘lme’ function in nlme R package^48^. Soil properties were treated as fixed effects, whereas sampling site was considered as a random effect.

To evaluate the effect of depth and soil properties on relative influence of bacterial community assembly processes, the pairwise comparisons of βNTI values were regressed against Euclidean distance matrices of depth and soil properties. The statistical significance of the resulting comparisons was determined by Mantel tests (‘mantel’ function in Ecodist R package^49^). Furthermore, we used multiple regression on distance matrices (MRM) approach^50^ to evaluate the relative importance of each of the soil properties on bacterial phylogenetic community assembly (βNTI). All the statistical analyses were performed using R software version 3.2.3^51^.

## Results and Discussion

### Soil properties

PCA ordination indicated the in terms of measured soil properties all the soil cores were distinct, except NY and SY cores (Fig. S1). A total of 93.7% of the variance was explained by the first two axes of PCA, with axis 1 and 2 explaining 54% and 39.7% of the total variance, respectively. PerMANOVA analysis further indicated a statistically significant effect of soil core sampling site on soil properties (R^2^ = 0.59, *P* < 0.001). Soil pH and electrical conductivity linearly increased along depth profile in each core (Fig. S2), with lowest pH and EC values being observed in PF core. These trends are in accordance with previous studies showing increase in soil pH^15,18,52^ and EC^31^ along depth profiles. The thick peat layer of PF core has resulted in detection of highest TOC, TN and GWC contents per unit soil mass in PF core, while other cores (TS, NY and SY) had relatively small amount of TOC, TN and GWC contents. Except pH and EC, the other parameters did not show consistent trend along depth profile in each core (Fig. S2).

### Quantitative PCR and dominant bacterial and archaeal taxa

**Figure 2:**
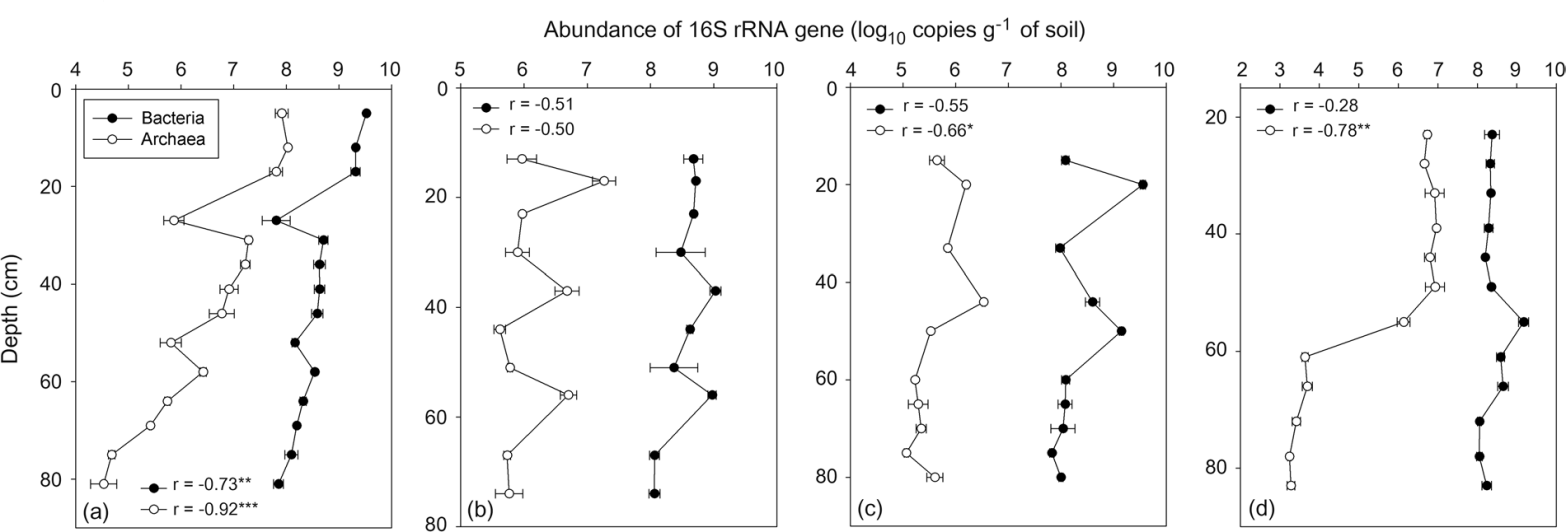
Bacterial and archaeal 16 S rRNA gene copies quantification along depth profiles of (**a**) TS, (**b**) NY, (**c**) SY, and (**d**) PF cores.

Vertical trends in the bacterial and archaeal abundances detected using qPCR varied by sampling site but they shifted in a similar manner with soil depth in TS, NY, and SY cores except for PF core (Fig. 2). In TS core, both bacterial and archaeal abundances declined with increasing depth with an abundance peak at surface soils. The high abundance of bacteria and archaea in surface soils compared to deeper soils has already been reported in previous studies^13,52^. The TS core has thinner organic layer and this might be the reason for observed strong negative correlation between bacterial and archaeal abundances and soil depth in TS core. In NY and SY cores, both bacterial and archaeal abundances were overall relatively uniform throughout the soil layers with a range of abundance fluctuations found at certain depths (Fig. 2). In PF core, bacterial abundance remained relatively constant across the depth, whereas archaeal abundance dramatically declined at a depth of 60 cm and maintained the abundance level down to the 80 cm. It is not clear why there is marked diminution in archaeal abundance at these depths. Regarding that methane concentration started increasing from a depth of 60 cm in PF core, the replacement of archaeal populations by methanogenic archaea at methane-rich soil layers may result in reduction of entire archaeal abundance.

**Figure 3:**
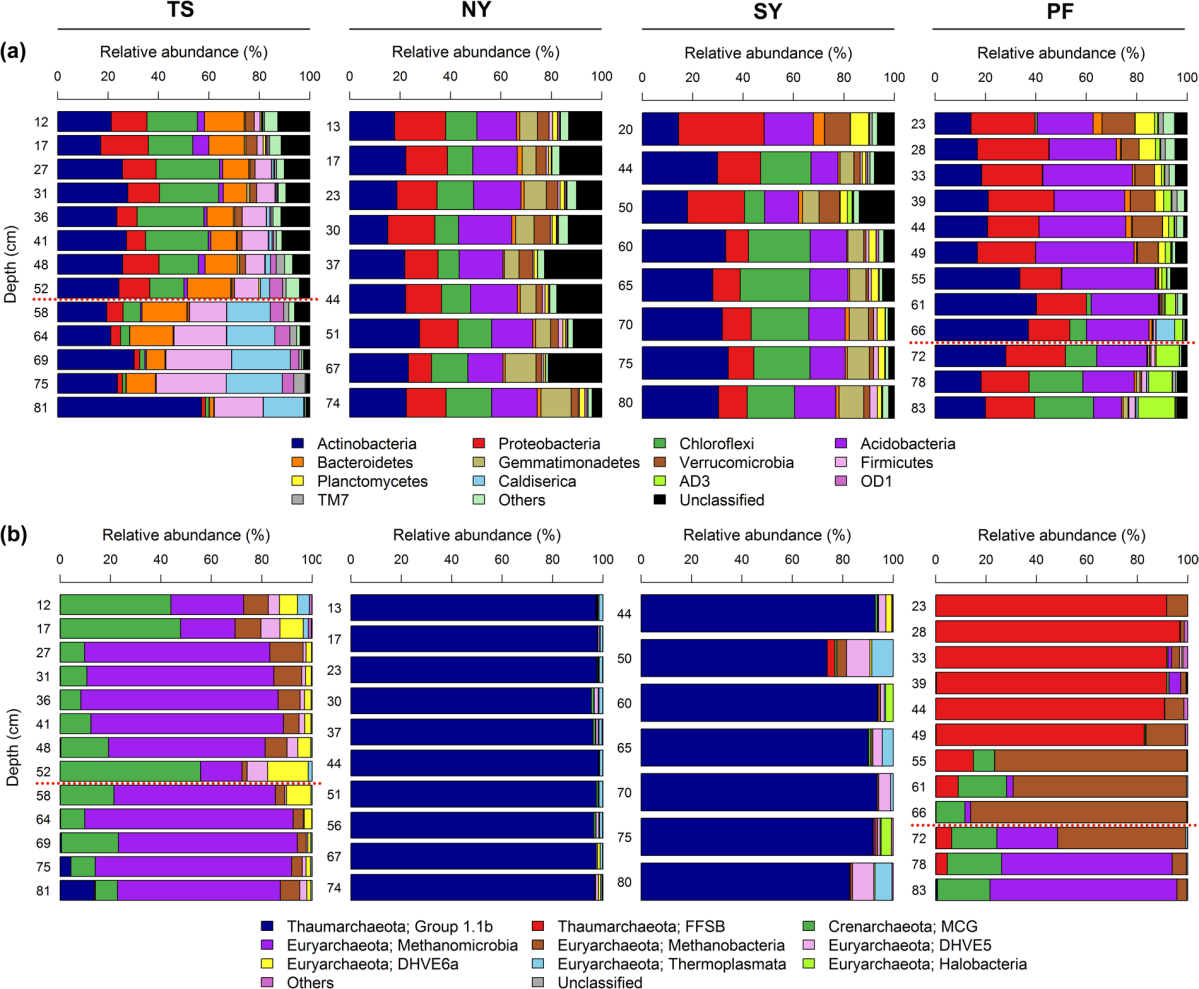
Relative abundance of dominant (**a**) bacterial (at phylum level) and (**b**) archaeal (at class level) taxa along depth profiles of soil cores. Permafrost table was represented by red dashed lines in TS and PF cores.

A total of 469,028 good quality 16 S rRNA gene sequences were obtained from 43 samples, and of which 369,582 and 99,446 sequences were of bacteria and archaea, respectively. The most abundant bacterial phylum across all samples was *Actinobacteria* (23.9% on average) followed by *Proteobacteria* (15.3%), *Acidobacteria* (14.7%), *Chloroflexi* (12%), *Firmicutes* (5%), *Bacteroidetes* (4.9%), *Verrucomicrobia* (3.6%), *Gemmatimonadetes* (2.9%), *Planctomycetes* (1.7%), *Caldiserica* (1.7%), AD3 (1.1%), OD1 (1.0%) and TM7 (0.9%) (Fig. 3a). The relative abundance of dominant bacterial phyla showed consistent depth related trend across all cores. Of these the relative abundance of *Proteobacteria*, *Acidobacteria*, *Bacteroidetes*, *Verrucomicrobia*, *Plactomycetes* and *Cyanobacteria* maintained at similar levels or decreased with depth (Fig. 3a). Our results are consistent with the findings of Kim *et al*.^18^, who also observed similar depth related trends in most these taxa in soil cores collected in Alaska. The decrease in relative abundance of *Proteobacteria* and *Bacteroidetes* with depth could be related to their copiotrophic life strategies because nutrient levels are normally greatest in top soils and deeper layers contain relatively less amounts of nutrients^53^. *Verrucomicrobia* and *Plactomycetes* were also shown earlier to dominate surface peat soils compared to deeper mineral soils^54^. The decrease in relative abundance of *Acidobacteria* could be related to increase in soil pH with depth, as several previous studies have showed that the relative abundance of *Acidobacteria* had a strong negative correlation with soil pH^55–58^. The decrease in relative abundance of photosynthetic bacterial phylum *Cyanobacteria* with depth could be related to reduced sunlight exposure with depth. Furthermore, consistent with prior studies, the relative abundance of *Actinobacteria* and *Firmicutes* increased with depth (Fig. 3a), which were commonly dominated in deeper mineral soils^15,31^. It has been also suggested that *Actinobacteria* and *Firmicutes* play important roles in anaerobic degradation of soil organic carbon at deeper depth levels^54^. Interestingly, *Chloroflexi*, which was shown earlier to dominate deeper soil layers^15,18,31^, showed contrasting trend with depth between cores (Fig. 3a). In TS core, there was a large proportion of *Chloroflexi* found throughout the active layer and its relative abundance became sharply reduced below 58 cm depth. In contrast, *Chloroflexi* was almost absent down to 55 cm depth in the active layer of PF core and became dominant in permafrost layer below a depth of 72 cm. These contrasting patterns suggest that some other unmeasured factors, either biotic or abiotic, may be involved in driving the relative abundance of *Chloroflexi*. Interestingly, an unknown *Chloroflexi* lineage (GQ396871_f) dominated deeper layers of PF core, whereas the majority of *Chloroflexi* in upper layers of TS core consist of family *Anaerolineaceae*, which are frequently found in petroleum-enriched areas and have potential of hydrocarbon degradation in anoxic conditions^59,60^. When representative sequences of major *Anaerolineaceae* OTUs were blast-searched against nr/nt database, they matched most closely to bacterial clone sequences recovered from benzene-contaminated river sediment, hydrocarbon-contaminated aquifer and oil reservoir (>99.0% identity with DQ463268, AF050569 and JQ668598, respectively). The greater relative abundance of phylum *Caldiserica* in permafrost layers of TS core also supports this because an unknown genus (EU266853_g) comprised the majority of this phylum and members belonging to this genus are often found in hydrocarbon-rich environments^61,62^. Another interesting finding was that the candidate bacterial phylum AD3 was dominant (~10%) in deeper permafrost layer of only PF core (Fig. 3a). The dominance of AD3 in deeper subsurface soils has been reported earlier^18,21,63^. Members of AD3 are known to be more abundant in deeper layers of mineral soil with low nutrient contents^63^. It corresponds to our results that SOC and TN contents dramatically declined below around permafrost table (80 cm depth) of PF core and soil texture also shifted from organic to mineral layers at the equivalent depth.

The most dominant archaeal taxa (at the class level) detected were different in each core, except in NY and SY cores (Fig. 3b). *Methanomicrobia* (61%) was the most dominant taxon detected in TS core (Fig. 3b), whereas FFSB (44%) was dominant in PF core (Fig. 3b). Both NY and SY cores showed similar taxonomic distribution with dominance of group 1.1b archaeal class (NY: 97% and SY: 88%) (Fig. 3b). The major depth related trends in the relative abundance of dominant archaeal taxa were observed only in TS and PF cores (Fig. 3b). The relative abundance of *Methanobacteria* decreased along depth profile in TS cores. Members of the *Methanobacteria* are hydrogenotrophic methanogens and more commonly found in upper soils^15,64,65^. The relative abundance of FFSB, which was the most dominant taxa in PF core, decreased with depth (Fig. 3b). The most obvious explanation for this finding is the increase in soil pH (4.34 to 5.62) along depth in PF core, as it is well documented that FFSB dominate acidic soils^66–68^, and their abundance decline quickly with increasing pH^69,70^. Furthermore, the relative abundance of *Methanomicrobia* increased with depth in PF core. Our findings support those of Deng *et al*.^15^, who found that the relative abundance of *Methanomicrobia* was higher in mineral permafrost layers.

**Figure 4:**
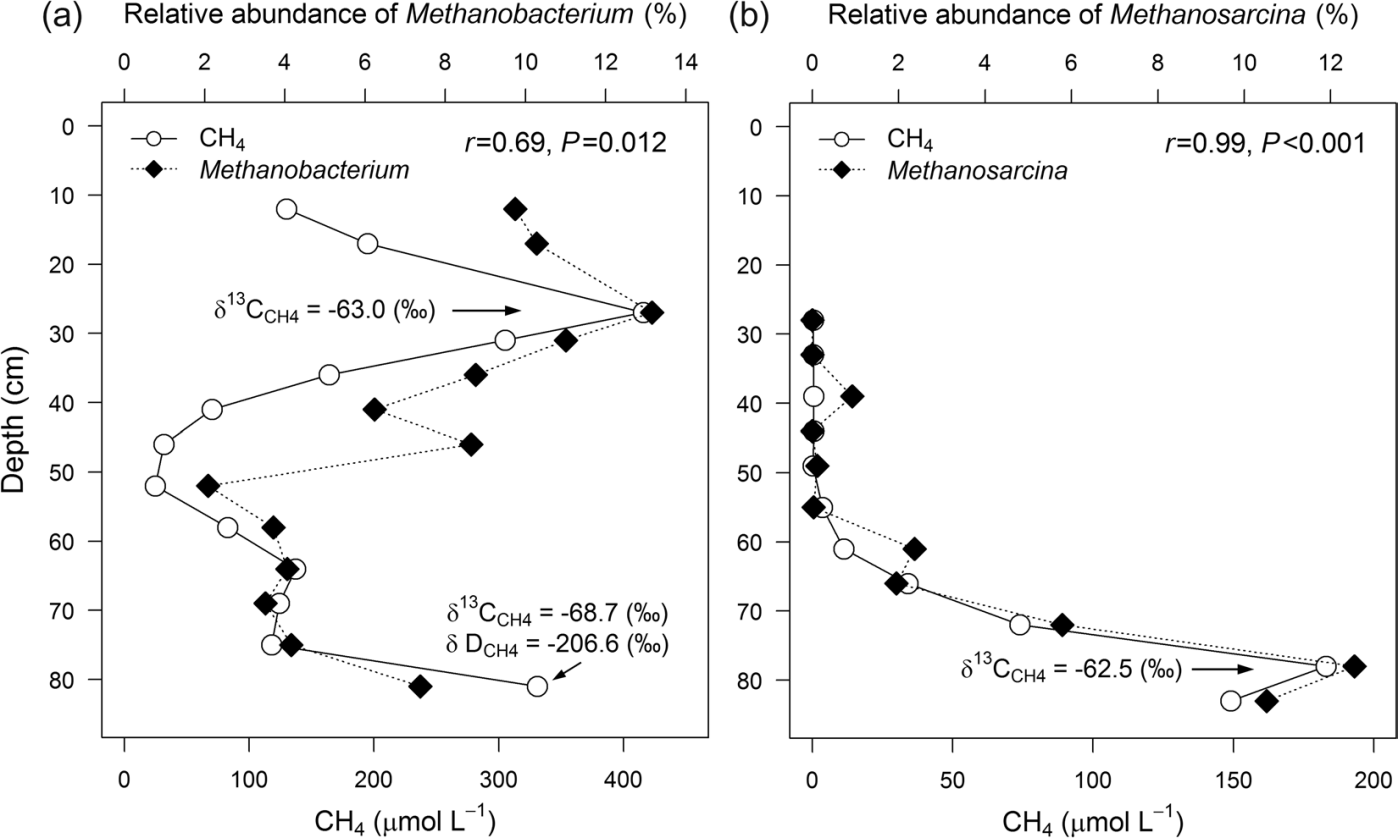
Correlation (Pearson) between the methane concentration and relative abundance of dominant methanogenic archaeal genera in (**a**) TS and (**b**) PF cores. Isotopic signatures of carbon and hydrogen (δ^13^C_CH4_ and δD_CH4_) at methane concentration peaks were retrieved from Byun *et al*.^32^.

The average methane concentrations of TS and PF core were much higher compared to other two cores with sharp increase in methane concentration at some depths (TS: at 27 and 81 cm depth; PF: at 78 cm depth) (Fig. S2). Stable isotope analysis revealed that methane at methane concentration peaks in these two cores is of microbial origin (see Fig. 3, Byun *et al*.^32^). Therefore, we further tested if there is any possible association between trapped methane and methanogenic archaeal taxa. We found significant correlations between methane concentrations and relative abundance of the dominant known methanogenic archaeal genera in both TS and PF cores (Fig. 4). In TS core, methane concentrations were significantly correlated with the relative abundance of hydrogenotrophic *Methanobacterium* (Fig. 4a), indicating that this hydrogenotrophic genus play important roles in subsurface methane generation under anaerobic conditions. The members of hydrogenotrophic methanogens were also found dominant in other studies on Arctic tundra soils^15,71^. It remains a moot point why methane and *Methanobacterium* relative abundance peak at both active and permafrost layer depths. Regarding the predominance of bacterial lineages associated with hydrocarbon degradation throughout the active layer, a methane peak at this depth may be ascribed to increased methanogenic degradation of hydrocarbons under poorly drained conditions formed at active layer of the TS core.

In PF core, the methane concentrations were strongly correlated with the relative abundance of acetoclastic *Methanosarcina* (Fig. 4b). This observation suggests that the methane peak in deeper permafrost layers of PF core was mainly formed due to acetoclastic methanogenesis. This is in agreement with the findings of other studies reporting the dominance of acetoclastic methanogenesis over hydrogenotrophic methanogenesis in peat soils^72–74^.

### Community composition

**Figure 5:**
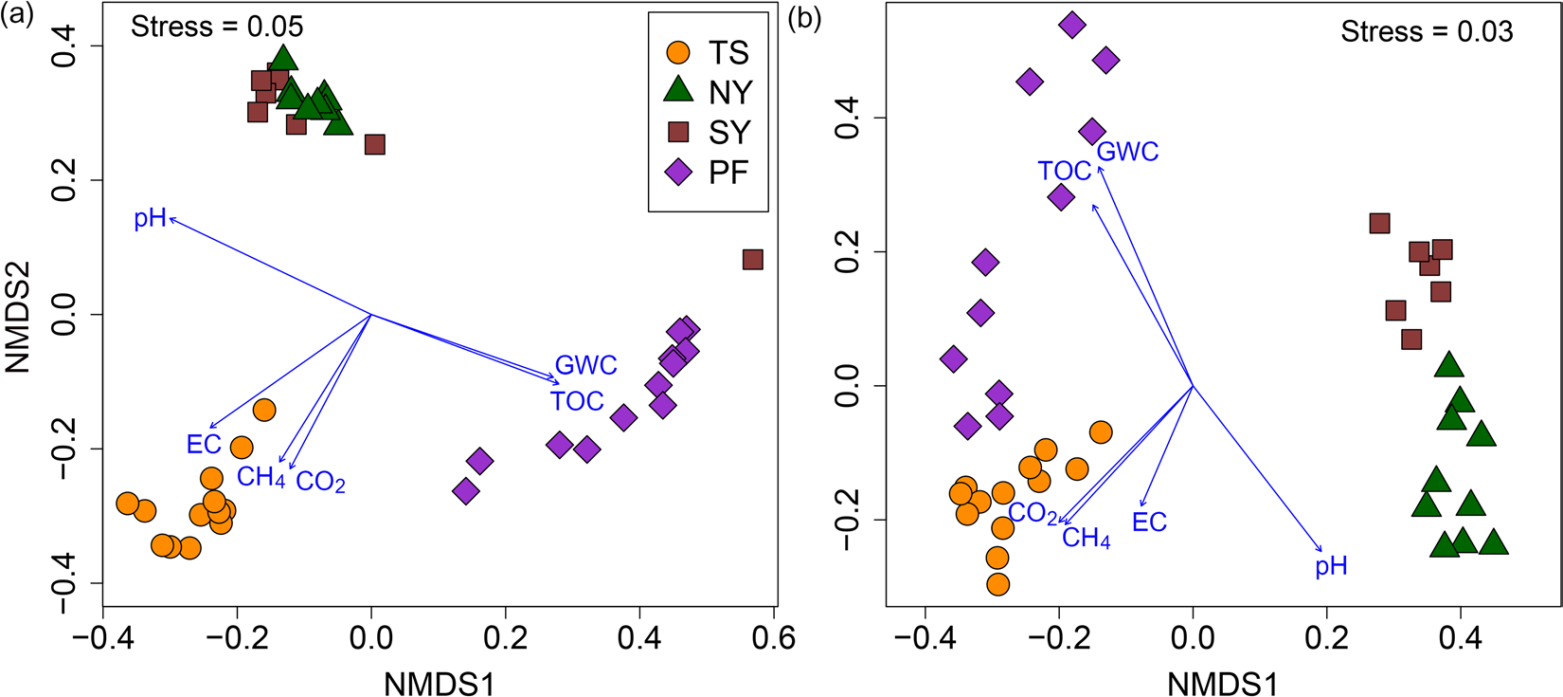
Non-metric multi-dimensional scaling plot of pairwise Bray–Curtis dissimilarities of (**a**) bacterial (**b**) archaeal communities. The vectors of significantly (*P* < 0.05) correlated variables are shown on the plot.

The NMDS ordination plot showed that the bacterial and archaeal community composition of soil cores was strongly clustered by sampling site (Fig. 5). The PerMANOVA analyses revealed that sampling site explained 48.7 and 54.5% of the variations in bacterial and archaeal community composition, respectively (*P* < 0.001, 9999 permutations). Moreover, certain dominant bacterial and archaeal OTUs also exhibited site-specific patterns (Fig. S3). Furthermore, we partitioned the explained proportion of the variation in bacterial and archaeal communities between soil properties, depth and geographical distance (Fig. S4). The largest fraction of the variation was explained by the geographically structured soil properties (35.6% for bacteria and 47.2% for archaea), of which the unique component of soil properties explained 13.4% and 10.8% of the variation in bacterial and archaeal communities, respectively. In comparison to soil properties, the unique component of geographical distance explained less variation - about 4.3% and 6.1% in bacterial and archaeal communities, respectively. The unique proportion of the variation explained by soil depth was very small and was only significant in explaining the variation in bacterial communities (Fig. S4). A large proportion of the variation (about 43.5% for bacteria and 35.4% for archaea) remained unexplained. We did not find significant influence of soil depth on bacterial and archaeal community composition (*P* > 0.05). This result is in contrast with the findings of Deng *et al*.^15^, who found that the largest fraction of variation in soil core bacterial community was explained by depth. However, in agreement with our result, Hu *et al*.^31^ found that the vertical distribution of bacterial community in permafrost soil core was primarily determined by physicochemical conditions rather than depth.

The environmental vector fitting analysis indicated that soil parameters such as pH, EC, GWC, and TOC significantly influenced the community composition of both bacteria and archaea (Fig. 5). It is well known that pH is one of the most important determinants of soil microbial community composition across various scales^12,55–57^. Soil pH has also been reported to influence the microbial community structure along the depth profile in permafrost soils^15,18,20^. Electrical conductivity^31^, GWC^21^ and total organic carbon^20^ are also reported earlier to influence the composition of the microbial communities with depth in permafrost soils. We also observed significant association between CO_2_ and CH_4_ concentrations and archaeal and bacterial community composition (Fig. 5). One of the possible reasons of this association could be that variations in soil physicochemical parameters along depth profile leads to change in community structure of bacteria and archaea, which might involve in key metabolic processes (such as acetoclastic methanogenesis or syntrophic acetate oxidation^75^) and influence the concentrations of CO_2_ and CH_4_ along the depth of soil cores.

### Diversity and assembly processes

**Figure 6:**
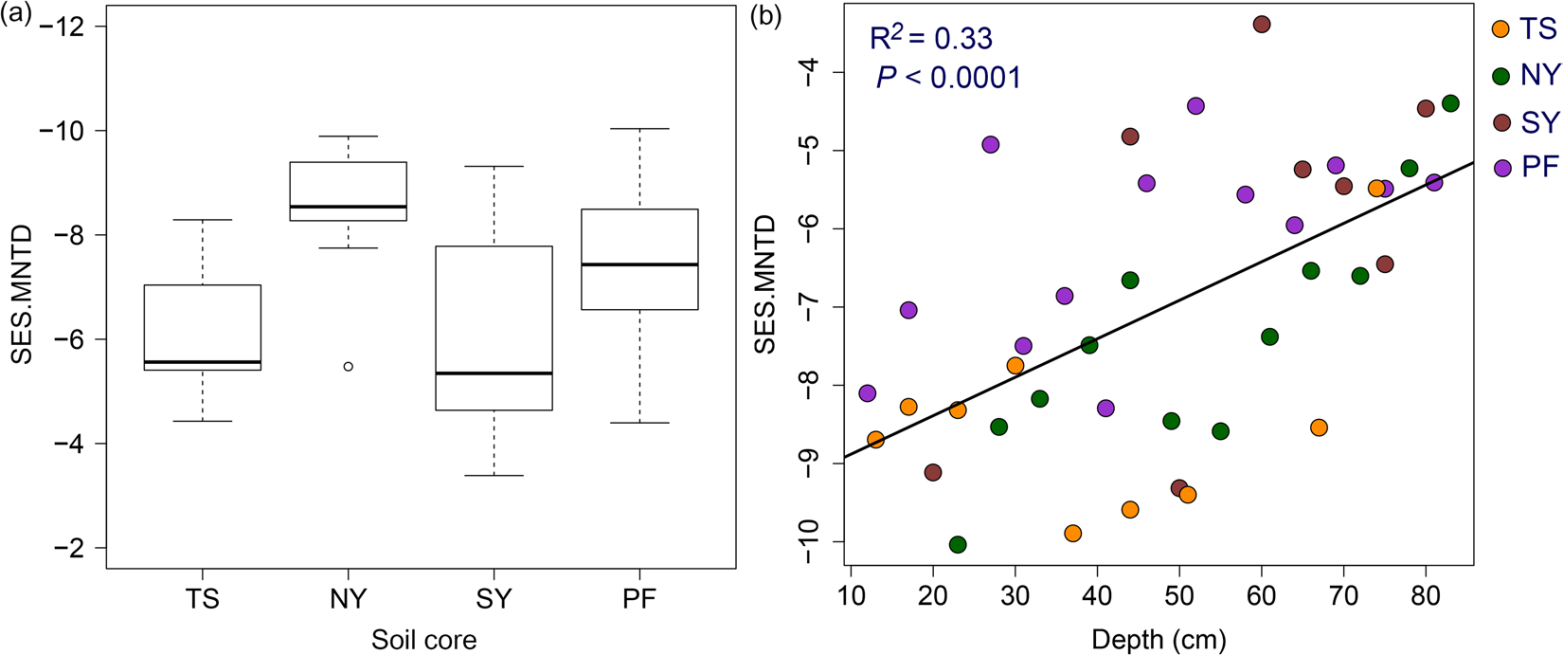
(**a**) Box plot showing variation in standardized effect size measure of mean nearest taxon distance (SES.MNTD) values of bacterial community along depth profiles of each soil core. (**b**) Relationship between depth and SES.MNTD across soil cores.

Bacterial diversity (Shannon index) decreased significantly with depth across all cores (Adj. R^2^ = 0.27, *P* < 0.001), and also influenced by soil properties (Table 1). The decrease in bacterial diversity along depth profile is commonly observed in several previous studies^15,18,52^. The change in environmental conditions with depth generate a strong ecological filter and this lead to decrease the bacterial diversity along depth profile, as many of the bacterial taxa are less likely to survive in deeper soil environment. The SES.MNTD values obtained using the null model were less than zero in all soil cores (Fig. 6a), indicating that the bacterial assemblages were phylogenetically clustered in each core along all depth. Our results are consistent with the findings of several previous studies in a wide range of environments^31,76,77^, which indicated that bacterial communities had a tendency to be phylogenetically clustered than expected by chance. The SES.MNTD values were positively correlated with depth across all cores (Fig. 6b). This indicates that bacterial community was shifted from phylogenetically more clustered assembly to less clustered assembly with increasing soil depth. It has been observed recently that bacterial lineages are more likely to be clustered in surface soils than subsurface soils^77^. Phylogenetically less clustered bacterial lineages in deeper soil layers could be resulted due to higher relative influence of stochastic processes in deeper soil layers than in surface soils.

**Table 1:** Linear mixed effects model summary table of effect of soil properties on bacterial diversity (Shannon index).

Fixed effect	Estimate	SE	df	t-value	p-value
Intercept	4.285	0.697	32	6.15	<0.0001
Depth	−0.012	0.003	32	−3.84	<0.001
pH	0.303	0.108	32	2.80	<0.01
EC	−0.003	0.001	32	−2.62	<0.05
TOC	0.025	0.001	32	2.55	<0.05
TN	−0.800	0.231	32	−3.46	<0.01
CH_4_	−0.001	0.0005	32	−2.48	<0.05

Abbreviations: EC = electrical conductivity; TOC = total organic carbon; TN = total nitrogen; CH_4_ = methane.

**Figure 7:**
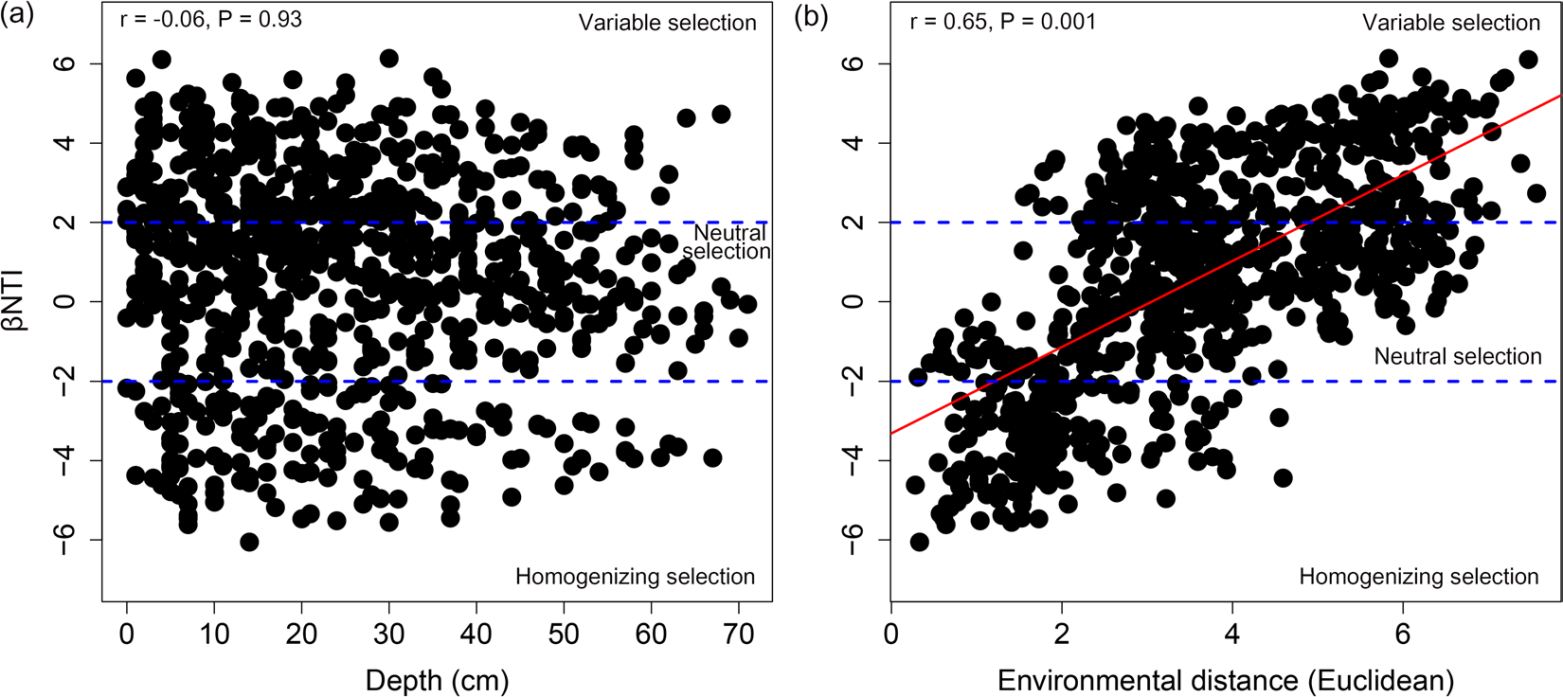
The relationships between β-nearest taxon index (βNTI) and (**a**) change in depth, and (**b**) environmental distances (Euclidean) between samples across soil cores. The statistically significant (Mantel test, 9999 permutations, *P* < 0.05) relationship is shown with solid red line.

We also calculated βNTI metric, which provides important insights about the community assembly processes. The turnover in βNTI metric among depth profiles across cores was significantly (Mantel *P* < 0.05) influenced with environmental distance, but not soil depth (Fig. 7). Among the soil properties measured, the turnover in βNTI metric was influenced by EC, CO_2_, GWC, pH, CH_4_ and carbon to nitrogen ratio (C:N) (Table 2). These results indicate that with progressive change in environmental distance, the community assembly processes shift from homogenous selection, to stochasticity, to variable selection. Overall, these results suggest that both deterministic and stochastic processes are important in governing the assembly of bacterial community along depth profiles and their relative influence vary with change in environment.

**Table 2:** Multiple regression on distance matrices (MRM) comparing the relationship between bacterial β-nearest taxon index (βNTI) and soil properties (Model: R^2^ = 0.54, *P* < 0.001)^a^.

Soil properties	Coefficient^b^
EC	0.89***
CO_2_	0.76***
GWC	0.75***
pH	0.58**
CH_4_	0.52**
C:N	0.43*

^a^The variation (R^2^) of βNTI that is explained by the remaining variables.

^b^The partial regression coefficients of the final model are reported from permutation test (nperm = 9999) with significance level alpha = 0.05. *P < 0.05; **P < 0.01; ***P < 0.001.

Abbreviations: EC = electrical conductivity; CO_2_ = carbon dioxide; CH_4_ = methane; GWC = gravimetric water content; C:N = carbon to nitrogen ratio.

Due to logistical limitations on sampling, we took only one soil core from each sampling site; however, this is insufficient to make any generalization about the depth profile of microbial communities in Alaskan soils. Therefore, the present study should be considered as a preliminary attempt for more in-depth future studies to provide a comprehensive understanding of depth profile of the microbial communities in Alaskan soils.

## Conclusions

In conclusion, our study demonstrates that the taxonomic composition of bacterial and archaeal communities of Alaskan soil cores is influenced by local environmental conditions (primarily soil pH, EC, GWC and TOC) rather than depth. However, bacterial and archaeal abundance and diversity were decreased along depth profile. The relative abundance of known methanogenic archaeal genera (*Methanobacterium* and *Methanosarcina*) was significantly correlated with the methane peaks detected at various depth levels in two soils cores. Bacterial phylogenetic community structure was shifted from more clustered to less clustered assembly with depth. Both deterministic and stochastic processes were involved in governing the assembly of bacterial community, and their relative influence varied strongly with change in environment rather than depth. Further studies on taxonomy and phylogeny of soil microbial communities along permafrost thaw gradient are necessary in order to have comprehensive understanding of the microbial ecology of permafrost soils.

### Data availability statement

All the 16 S rRNA gene sequence data used in this study are deposited to the Sequence Read Archive (SRA) at NCBI under the accession number SRP124819.

## Electronic supplementary material

Supplementary Information
