# Supplementary material for: Variations in bacterial and archaeal communities along depth profiles of Alaskan soil cores
Source: Sci Rep. 2018 Jan 11;8:504. doi: 10.1038/s41598-017-18777-x (PMC5765012; doi:10.1038/s41598-017-18777-x)
Supplement: Supplementary file 1 — Supplementary Information [file 41598_2017_18777_MOESM1_ESM.doc]

**Supplementary information**

**Variations in bacterial and archaeal communities along depth profiles of Alaskan soil cores**

Binu Mani Tripathi^1 a^, Mincheol Kim^1 a^, Yongwon Kim^2^, Eunji Byun^3^, Ji-Woong Yang^3^, Jinho Ahn^3^ and Yoo Kyung Lee^1*^

^1^Korea Polar Research Institute, Incheon 21990, Republic of Korea

^2^International Arctic Research Center, University of Alaska, Fairbanks, Alaska, USA

^3^School of Earth and Environmental Sciences, Seoul National University, Seoul, Republic of Korea

^a^These authors equally contributed to the work

***Correspondence:** Yoo Kyung Lee, E-mail: [yklee@kopri.re.kr](mailto:yklee@kopri.re.kr)


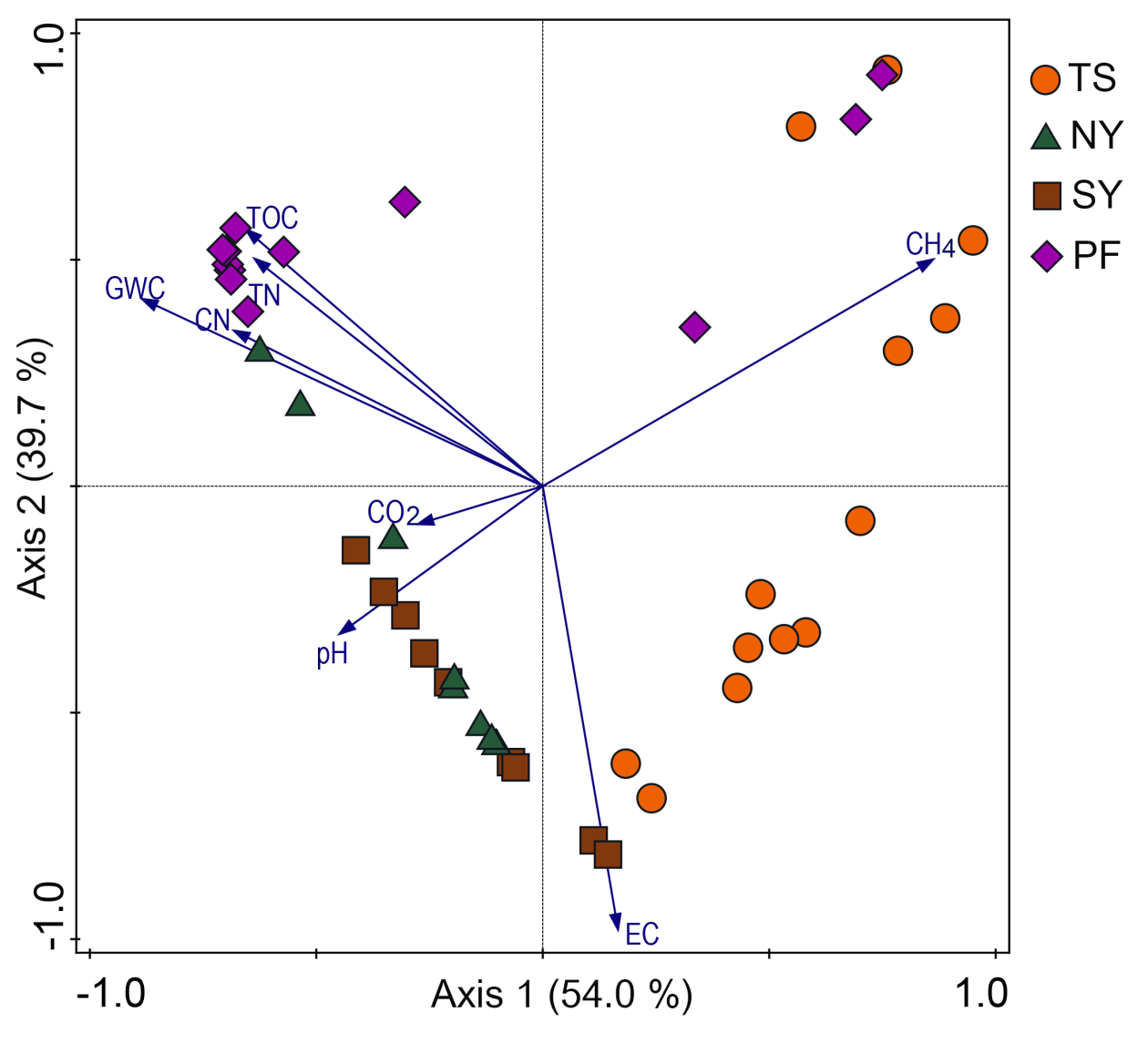


**Fig. S1.** Principal components analysis (PCA) of depth profiles across sites based on soil properties only. EC= electric conductivity, TOC= total organic carbon, TN= total nitrogen, CN= carbon and nitrogen ratio, and GWC= gravimetric water content.


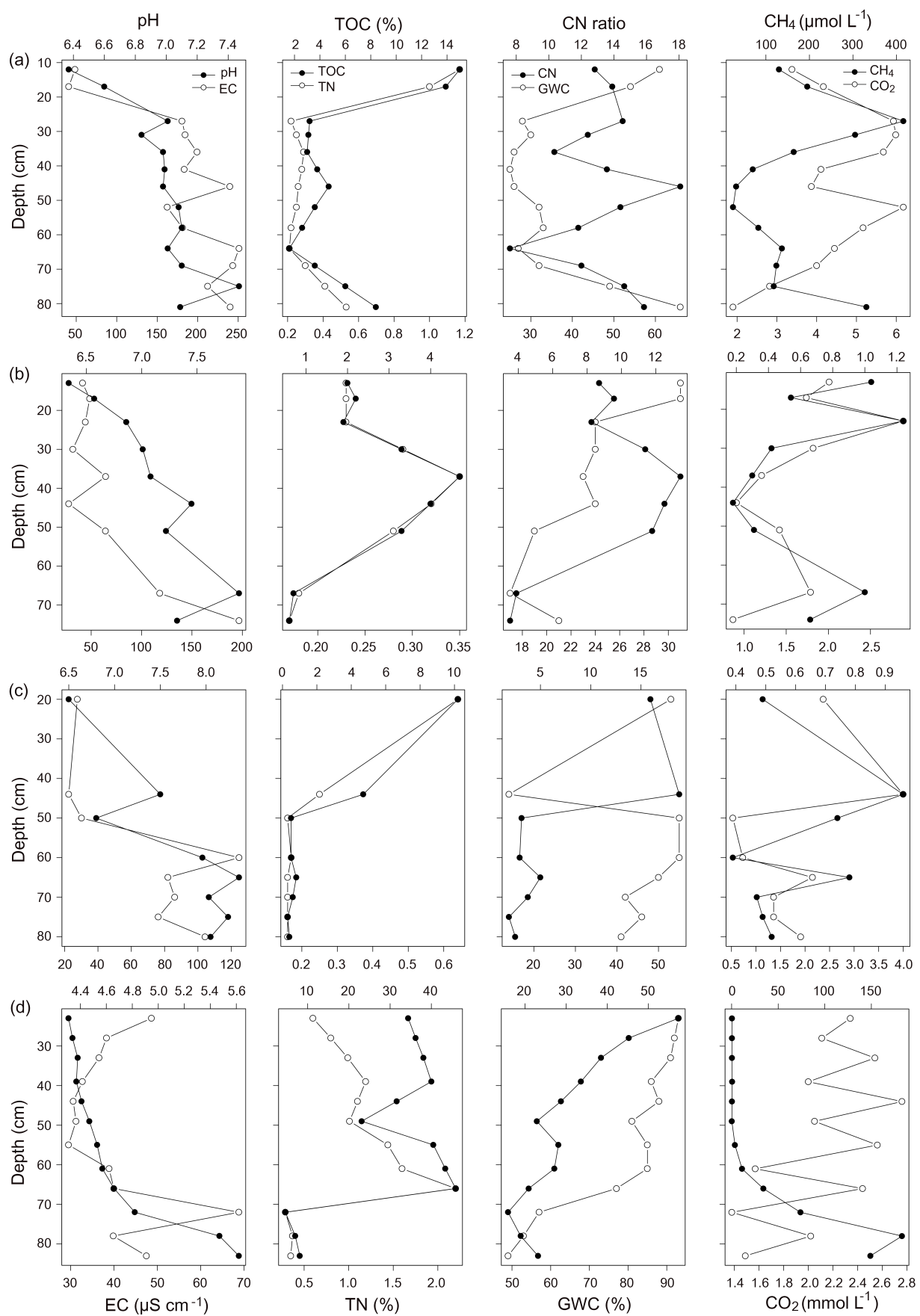


**Fig. S2.** Soil properties along depth profiles of (a) TS, (b) NY, (c) SY, and (d) PF cores. EC: electrical conductivity, TOC: total organic carbon, TN: total nitrogen, CN ratio: carbon to nitrogen ratio, and GWC: gravimetric water content. Depth profiles of TOC, TN, CN ratio, GWC were obtained from Byun et al. (2016).


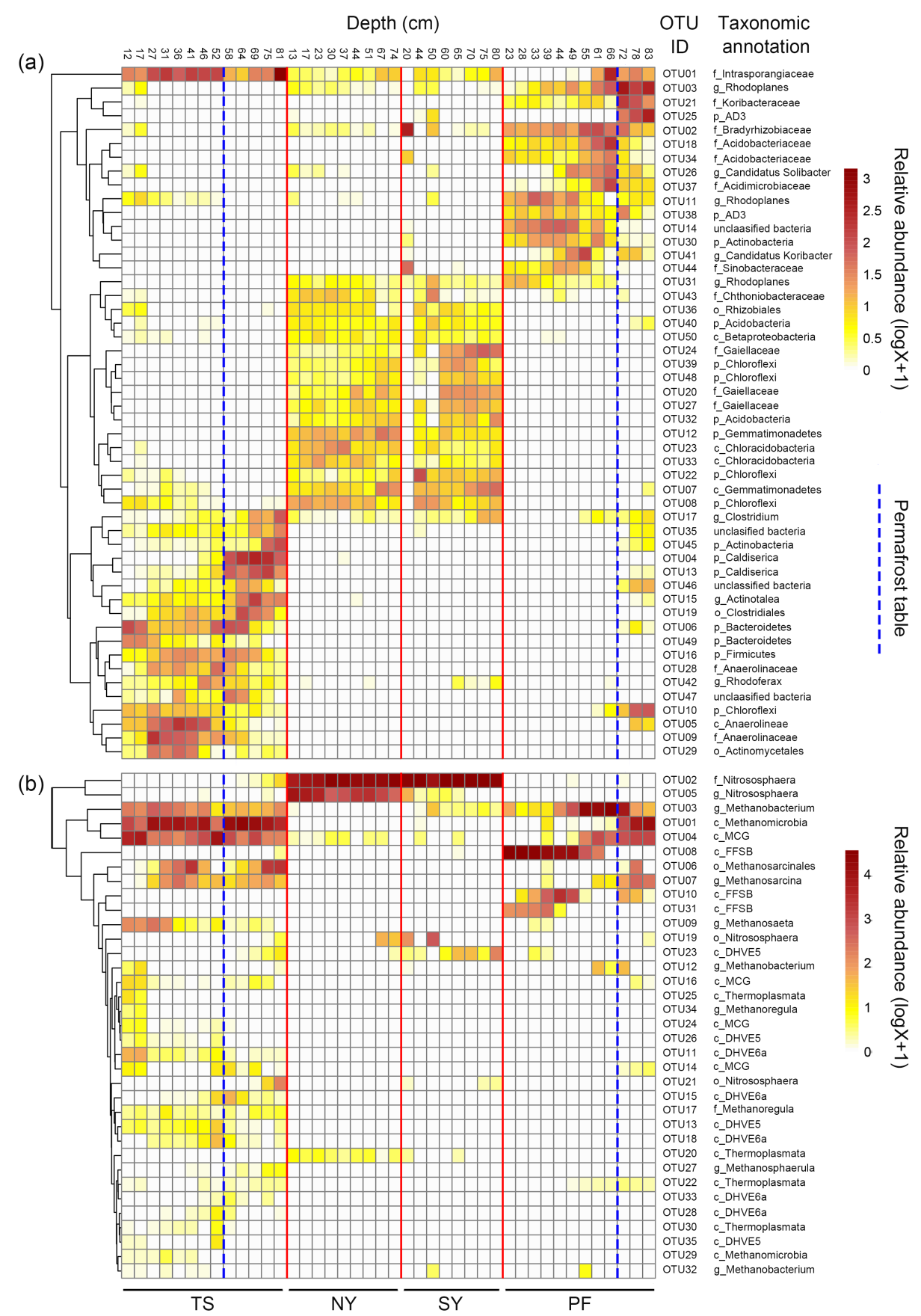


**Fig. S3.** The heat map shows the relative abundances of (a) 50 most abundant bacterial and (b) 35 most abundant archaeal OTUs along depth profiles of soil cores.


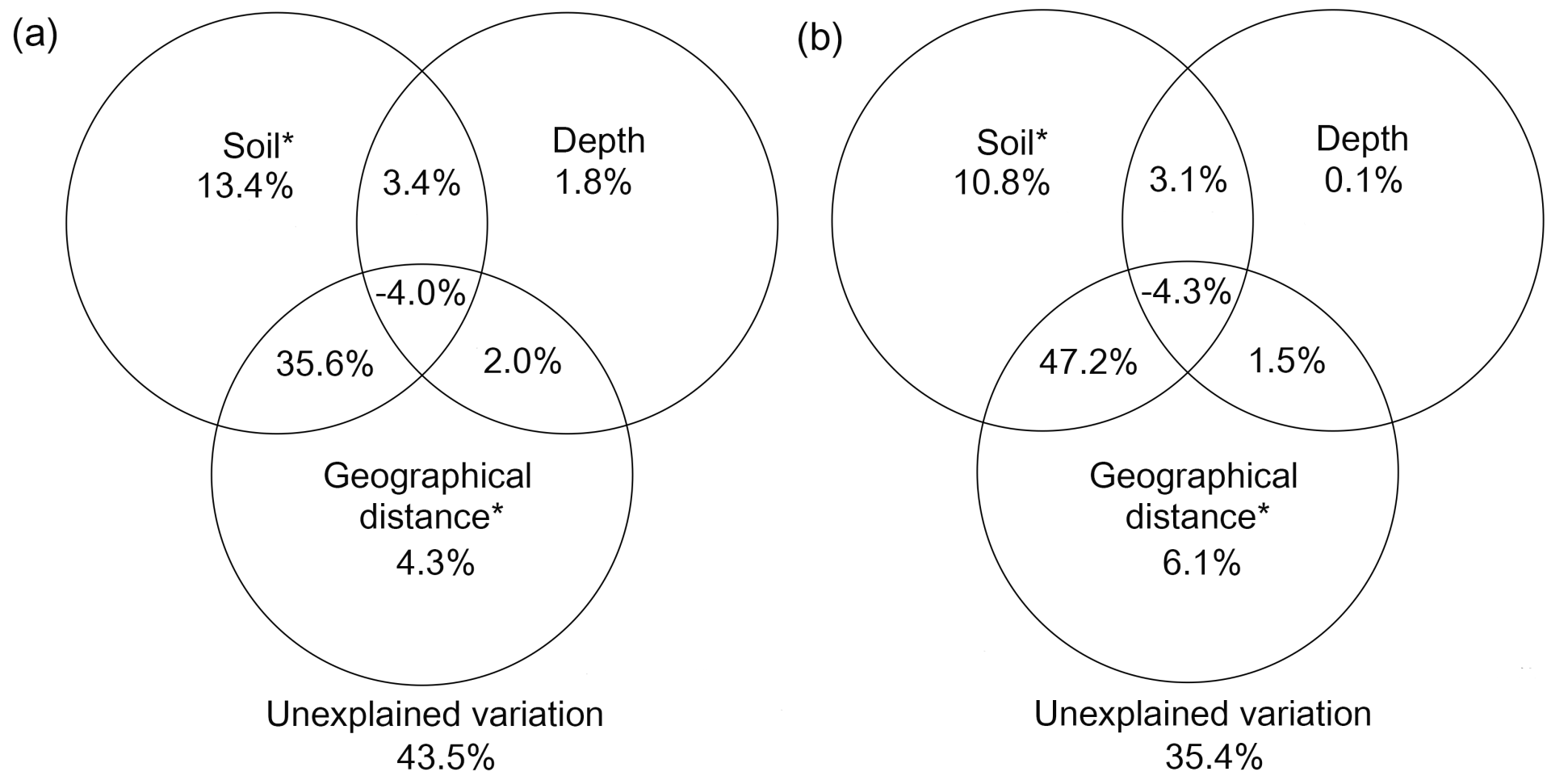


**Fig. S4.** Percentage of variation explained by soil properties, depth and geographical distance of (a) bacterial and (b) archaeal community compositions. **P* < 0.05.
